# Order of removal of conventional and nonconventional introns from nuclear transcripts of *Euglena gracilis*

**DOI:** 10.1371/journal.pgen.1007761

**Published:** 2018-10-26

**Authors:** Natalia Gumińska, Magdalena Płecha, Bożena Zakryś, Rafał Milanowski

**Affiliations:** Department of Molecular Phylogenetics and Evolution, Institute of Botany, Faculty of Biology, Biological and Chemical Research Center, University of Warsaw, Warsaw, Poland; University of Dundee, UNITED KINGDOM

## Abstract

Nuclear genes of euglenids and marine diplonemids harbor atypical, nonconventional introns which are not observed in the genomes of other eukaryotes. Nonconventional introns do not have the conserved borders characteristic for spliceosomal introns or the sequence complementary to U1 snRNA at the 5' end. They form a stable secondary structure bringing together both exon/intron junctions, nevertheless, this conformation does not resemble the form of self-splicing or tRNA introns. In the genes studied so far, frequent nonconventional introns insertions at new positions have been observed, whereas conventional introns have been either found at the conserved positions, or simply lost. In this work, we examined the order of intron removal from *Euglena gracilis* transcripts of the *tubA* and *gapC* genes, which contain two types of introns: nonconventional and spliceosomal. The relative order of intron excision was compared for pairs of introns belonging to different types. Furthermore, intermediate products of splicing were analyzed using the PacBio Next Generation Sequencing system. The analysis led to the main conclusion that nonconventional introns are removed in a rapid way but later than spliceosomal introns. Moreover, the observed accumulation of transcripts with conventional introns removed and nonconventional present may suggest the existence of a time gap between the two types of splicing.

## Author summary

The existence of conventional spliceosomal introns in genes of eukaryotic organisms is a well-known theorem. However, genes of the unicellular algae group, euglenids, contain also another type of introns, so-called nonconventional ones. They lack canonical borders, a feature most characteristic for conventional introns and form a stable secondary structure bringing together their ends. Along with the increasing popularity of whole genome studies, nonconventional introns were also disclosed in the genes of other protists, diplonemids. In this study we were particularly interested which introns–conventional or nonconventional–are removed earlier from euglenids’ pre-mRNA. To track this process we analyzed transcripts of the two *Euglena gracilis* genes. The relative order of intron excision was compared for pairs of introns belonging to different types. We also surveyed thousands of intermediate products of splicing using the Next-Generation Sequencing system. Summarizing the results of both experiments, we proved that spliceosomal introns are removed at an earlier stage of pre-mRNA maturation than nonconventional ones.

## Introduction

Nuclear genes of eukaryotes contain introns which are removed from pre-mRNA in a splicing process catalyzed by the spliceosome–a ribonucleoprotein complex assembled from five small nuclear RNAs (snRNA) and a range of proteins. Intron removal begins co-transcriptionally–when the pre-mRNA synthesis on the DNA template by RNA polymerase II is yet to be completed [1–5]. This is possible due to the transcribed introns’ sequences becoming available for further processing as the transcription machinery moves down the DNA template along the reading frame. Interestingly, many splicing factors are prepositioned on the polymerase II C-terminal domain, which accelerates their action [6,7]. Furthermore, splicing of adjacent introns often proceeds collinearly with the progress of transcription. Therefore, introns synthesized earlier tend to be removed before the following introns, according to the ‘first-come-first-served’ model [8,9]. However, the order of intron removal is not always consistent with the arrangement in which they appear, both in the gene and in the transcript [10–13]. Several factors influence the directing of intron removal. First, the excision of one intron may be dependent on the removal of another one. Secondly, introns can be removed at different rates; in turn, excision rate depends on many factors such as intron length, exon length, the composition of intronic and exonic splicing enhancers and silencers or on the secondary structure of the intron itself [5,12]. Thus, many introns are spliced out much later than would be expected from their position in the transcript–often in the post-transcriptional splicing process located elsewhere in the cell nucleus. Non-collinear removal of introns from pre-mRNA is observed in transcripts which can undergo alternative splicing. This process takes place when the removal of the first alternative intron is delayed. The delay adds processing time, which allows the detection of splice signals for further alternative introns. It has been observed that in alternatively spliced transcripts, constitutive introns are mainly co-transcriptionally removed, whereas alternative introns–post-transcriptionally [3,5].

The rules mentioned above describe the maturation of regular eukaryotic pre-mRNA containing merely typical, conventional, spliceosomal introns. However, this is not the only intron type to be found in eukaryotic protein-coding genes. Nuclear genes of some euglenozoans (supergroup Excavata) harbor atypical, nonconventional introns which are not observed in the genomes of other eukaryotes. The Euglenozoa group comprises: (1) kinetoplastids, including parasites causing diseases such as the Chagas disease (*Trypanosoma cruzi*), African trypanosomiasis (*Trypanosoma brucei*) or leishmaniasis (many species of *Leishmania*), (2) euglenids, including a large group of photosynthetic species with secondary plastids of green algal origin, (3) diplonemids, free-living phagotrophs, common predators dominating the marine environment and (4) poorly researched symbiontids, free-living flagellates inhabiting low-oxygen marine sediments [14]. Genomes of parasitic kinetoplastids are strongly reduced and contain hardly any introns at all. However, these organisms have a functional spliceosome involved in the addition of splice-leader sequences to mRNAs by *trans*-splicing [15]. On the contrary, genomes of euglenids [16] contain a wide repertoire of introns: conventional (spliceosomal) as well as nonconventional ones, removed from the pre-mRNA through an unknown mechanism. Recently, the nonconventional introns have also been found in marine diplonemids–one of the most common eukaryotic groups in the oceans [17,18]. Single-cell genome sequencing of ten diplonemid species isolated from waters around the coasts of California revealed that their genes contain numerous nonconventional introns. What is more, as of yet no spliceosomal introns were detected in the analyzed genes [19]. However, the nonconventional introns of euglenids remain the most extensively studied so far [20–27]. Nonconventional introns present in nuclear genes of euglenids (aside from the spliceosomal ones) do not contain conserved borders, characteristic for spliceosomal introns, or the sequence complementary to U1 snRNA at the 5' end [28]. To date, merely about 100 introns of this type have been described. The data are scarce, because no complete genomic sequence is known for any euglenid representatives. Currently the *Euglena gracilis* (model euglenid species) genome sequencing project is in progress but available data are still far from satisfactory. The reason for that is the unexpectedly large size of the genome (about 2 Gbp) and high level of repetitive sequences. Only a few genes were annotated so far, but the presence of two types of introns was confirmed on the genomic level [29]. However, a complete survey of nonconventional introns on the whole-genome should be continued. Nonconventional introns are known to form a stable secondary structure, bringing together both exon/intron junctions, nevertheless this conformation does not resemble the form of self-splicing or tRNA introns and is only conserved to a small extent. Hitherto, only the base pairing between +4, 5, 6 nucleotides at the 5' end and -8, 7, 6 at the 3' end has been noticed in the majority of them. The nucleotides at exon/intron junctions are also only slightly conserved: Y/R usually appear at the 5' junction and Y/R at the 3', although the deviations from this model are quite common [21]. Due to the presence of inverted repeats at the ends of the introns, which are responsible for bringing their adjacent ends close to each other, it has been suggested that introns of this type may originate from non-autonomous transposons similar to MITEs (Miniature Inverted-repeat Transposable Elements) and propagate throughout the genomes as mobile genetic elements. In the genes studied so far, frequent insertions of nonconventional introns at new positions have been observed, whereas conventional introns have been either found at the conserved positions, or simply lost [21]. The special type of introns in nuclear genes of euglenids are so-called intermediate introns, which have some features of both conventional and nonconventional introns [20,24]. However, it seems that majority of them are rather typical conventional or nonconventional introns and their similarity to the second type is quite accidental [21,22].

There are nuclear genes of euglenids in which exclusively nonconventional introns (e.g. *rbcS*), solely conventional (e.g. *tubG*), or introns of both types (e.g. *tubA*, *gapC*) occur. It is also worth mentioning that some nuclear transcripts of euglenids undergo *trans*-splicing, in which the splice-leader sequence deriving from the SL-RNA is added to the 5' end of a transcript in place of an outron, a semi-intron containing the branch point A, polypyrimidine tract and the 3' splice site AG [27,30]. The studies aimed at comparison of the removal order of the *trans*-spliced outron and the first *cis* intron were performed only for genes harboring exclusively either nonconventional introns (*rbcS*) or conventional ones (*tubG*). According to previous reports, in the *rbcS* gene, nonconventional *cis*-splicing occurs earlier than conventional *trans*-splicing [26], while in the *tubG* gene, conventional *trans*-splicing takes place before conventional *cis*-splicing [20]. These results suggest that the process of nonconventional intron removal is rapid and precedes the removal of an outron. Furthermore, the addition of the splice-leader seems to likewise take place before the conventional splicing. However, the order of intron removal has never been examined for transcripts containing both types of introns. Therefore, in this work, we present the results of such analysis for two genes (*tubA* and *gapC*). The relative order of intron removal was compared for pairs of introns belonging to different types. Additionally, intermediate products of splicing were analyzed using the Next Generation Sequencing (NGS) technology of PacBio SMRT system (Pacific Bioscience) generating long NGS reads (on average up to 15–20 kbp long). Interestingly, the results obtained differed from previous findings.

## Results

### The relative order of intron removal in the *tubA* and *gapC* transcripts

**Fig 1:**
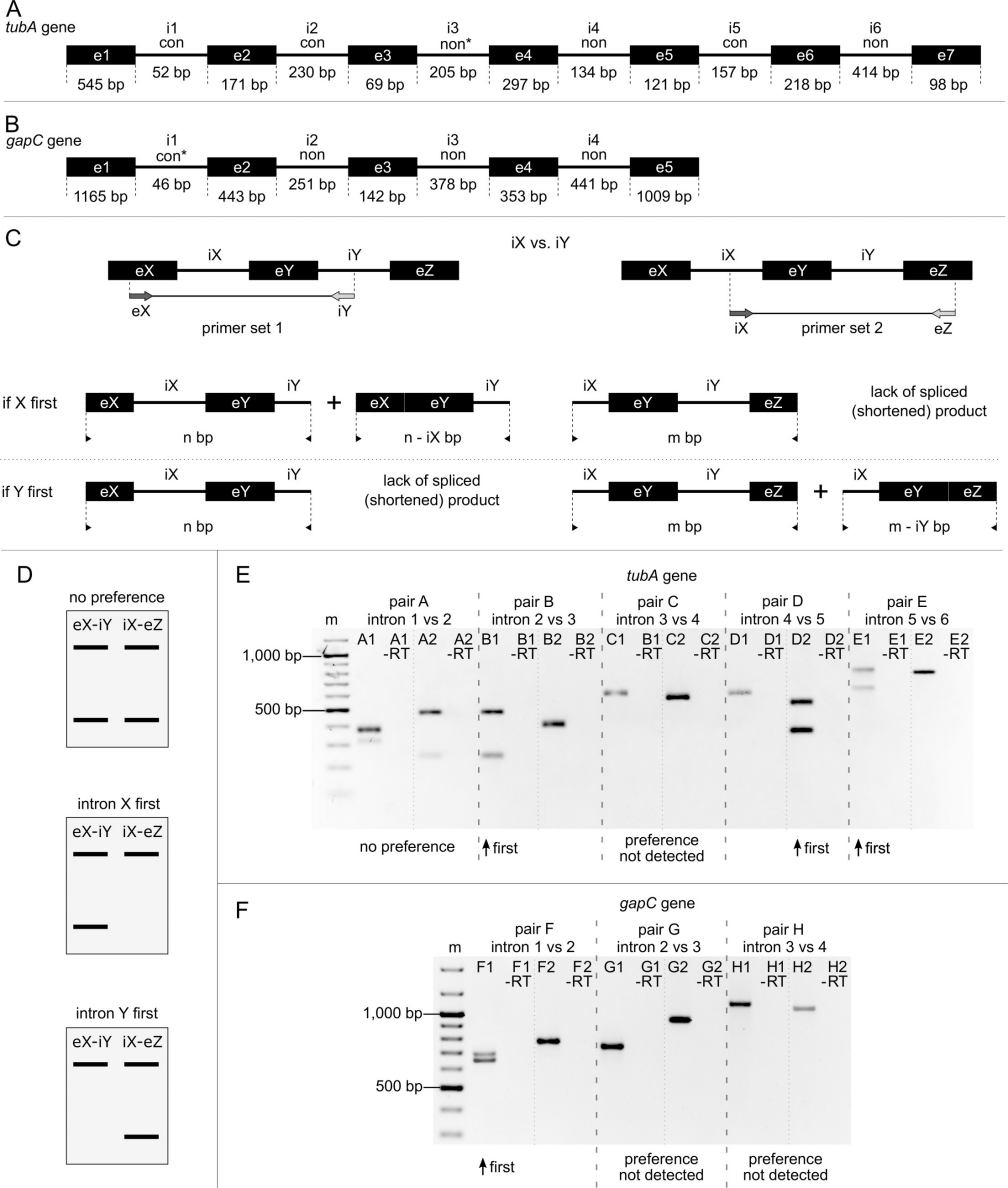
Pairwise comparisons of adjacent introns removal. (A) Schematic map of the *tubA* gene. (B) Schematic map of the *gapC* gene. Boxes and lines represent, respectively, the exons and the introns of the *gapC* and *tubA* transcripts (not to scale). The symbols used are as follows: e–exons, i–introns, con–conventional, non–nonconventional, asterisk–introns that exhibit intermediate features. (C) The two different pairs of exonic (e) and intronic (i) primers used to determine the splicing order of two adjacent introns (iX, iY) and the different types of molecules expected if a given intron is spliced before the other, or vice versa (the arrows represent primers). (D) The possible images of an agarose gel reflecting, respectively, no preferences, preferential removal of the intron X before Y, vice versa. (E-F) RT-PCR products obtained with various primer sets designed for the (E) *tubA* gene, (F) *gapC* gene, respectively. The first lane of each gel contains the DNA ladder GeneRuler 100 bp plus (Thermo Scientific), the following lanes contain PCR products (one primer set in each lane) as well as corresponding negative controls (-RT reactions). All obtained products were gel purified and sequenced to validate their origin. The dashed lines were introduced as visual aid to separate products obtained with each two compared primer sets and do not indicate fragments cropped from different gels.

The *tubA* gene of *E*. *gracilis* contains six introns (Fig 1A), three conventional (positions 1, 2 and 5), two nonconventional (positions 4 and 6) and one intermediate intron at position 3. This intron possesses GC-AG ends characteristic for conventional introns, however, if its boundaries were to be defined by only one nucleotide upstream (without disturbing the protein coding potential), then the secondary structure of this intron (on the RNA level) would be consistent with the model of nonconventional introns. Moreover, the intron occurs at the position unique for *E*. *gracilis*. This observation suggests that this intron is rather removed in the nonconventional manner [21]. The *gapC* gene of *E*. *gracilis* contains four introns (Fig 1B). The first of them was defined as an intermediate one, as its boundaries are typical for spliceosomal introns, albeit base pairing of its ends is observed. On the other hand, it seems that this is a conventional spliceosomal intron, as it appears at the position where typical conventional introns are present in relatives of *E*. *gracilis*. Furthermore, its secondary RNA structure does not resemble that of nonconventional introns [22]. The remaining three introns in the *gapC* gene are nonconventional ones.

An analysis of the relative order of intron removal was performed for all overlapping intron pairs in both genes–*tubA* and *gapC*, respectively [11]. Two sets of primers were designed for each intron pair; in the first set the forward primer was complementary to the exon preceding the first intron, while the reverse primer–to the second intron in the examined pair. In the second set the forward primer was complementary to the first intron and the reverse primer to the exon following the second intron (Fig 1C, S1 Fig). The primer pairs were used in a PCR reaction with cDNA obtained by reverse transcription with random hexamers. Two bands indicate the presence of two types of pre-mRNA, one with both introns (full-length) and the second with one intron removed. Two bands in both reactions indicate lack of preference of intron removal, while the presence of two bands in one reaction and just one band in the second indicates the preferred order of intron removal (Fig 1D).

The results obtained for both analyzed genes were compatible. In the case of a pair of conventional introns in the *tubA* gene, two bands were present in both reactions (Fig 1E, pair A). On the other hand, in the case of pairs of nonconventional introns (including also the intermediate intron) in both genes, only singular, longer products corresponding to the unspliced pre-mRNA were present (Fig 1E and 1F, pairs C, G and H). Whereas, when introns of different types were taken into consideration, if the primer complementary to conventional intron yielded a product, it always contained the neighboring nonconventional intron. However, if a nonconventional intron was present, two bands, corresponding to the pre-mRNAs before and after conventional splicing were observed (Fig 1E and 1F, pairs B, D, E and F).

### Order of outron and first intron removal in the *rbcS* transcript

**Fig 2:**
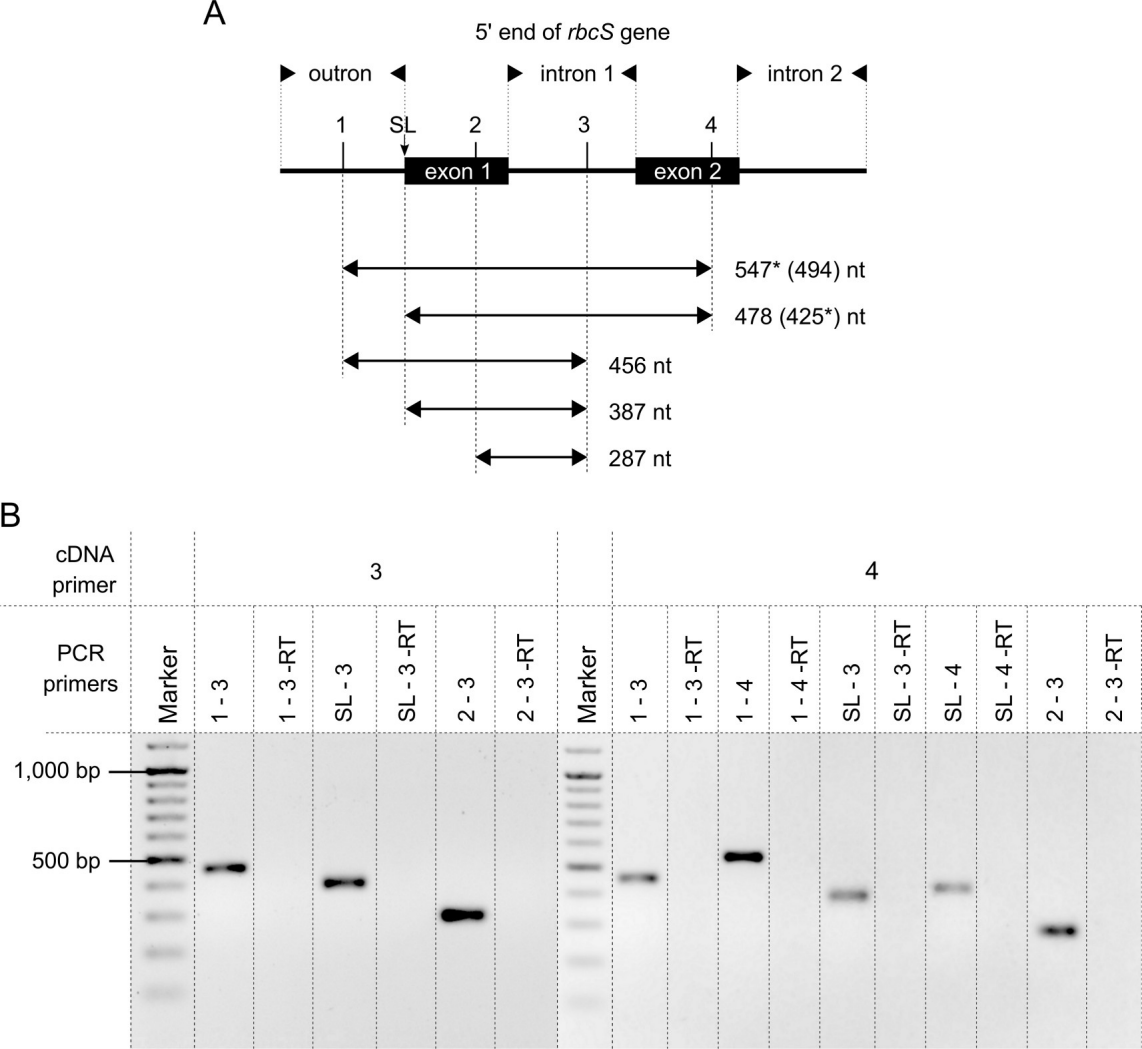
Order of removal of the outron and the first intron in the *rbcS* transcript. (A) Schematic map of the 5’ end of the *rbcS* gene (not to scale). Boxes and lines represent, respectively, the exons and the sequences spliced out from pre-mRNA (outron and introns). The exons are labelled inside the boxes. The intervening sequences are labelled above the map (outron and two adjacent introns). The relative positions of the splice leader (SL; indicated with a vertical arrow) and the primers used in this study (1–4) are pointed out below the captions of sequence regions. The positions of the RT-PCR products are indicated by horizontal arrows with their predicted sizes. In parentheses, the sizes of intron-less products are shown. If two products of different length were possible (1–4; SL-4), the length of the obtained one was marked with an asterisk. (B) RT-PCR products obtained with various primer sets used in this study. Total RNA was extracted, treated with DNase, reverse transcribed, amplified and electrophoresed through an agarose gel. All obtained products were further purified and sequenced to validate their origin. RT primers are listed in the upper row of the table, PCR primers corresponding to individual products are listed in the bottom row. Two agarose gels (corresponding to RT primers, respectively) were merged within the table. The first lane of each gel contains the DNA ladder GeneRuler 100 bp plus (Thermo Scientific), whilst the following lanes contain PCR products (one primer set per each lane) and corresponding negative controls (-RT reactions).

Results obtained for *tubA* and *gapC* transcripts contradicted the data in the literature which indicates that nonconventional splicing occurs before the conventional one [26]. Therefore, the experiment first performed by Tessier et al., which demonstrates the comparison of the order of outron and first nonconventional intron removal, has been repeated by our team. The 5' end structure of the *rbcS* gene and the positions of primers used in the study are shown in Fig 2A. The cDNA synthesized in a reverse transcription reaction with primers binding in positions 3 and 4 was used as a template for upcoming PCR reactions. Control reactions with primer sets located in positions 1–3, 2–3 and SL-4 yielded results identical to those described previously. However, the results allowing for the inference of the splicing order were different (Fig 2B). For primer set 1–4, one band corresponding to an unprocessed pre-mRNA with an outron and nonconventional intron was obtained (in the previous study two products were reported and they were not confirmed by sequencing). Interestingly, in reactions with the SL-3 primer set, the PCR products were also obtained indicating the presence of the *rbcS* transcript with the splice leader attached instead of an outron, and with the first nonconventional intron still unspliced (no product had been found in the previous report). This result proves that conventional *trans*-splicing may occur before nonconventional *cis*-splicing. Furthermore, the outcome is consistent with the results obtained for the *tubA* and *gapC* transcripts.

### The analysis of *tubA* and *gapC* pre-mRNA splicing intermediates inferred from PacBio NGS sequencing

**Fig 3:**
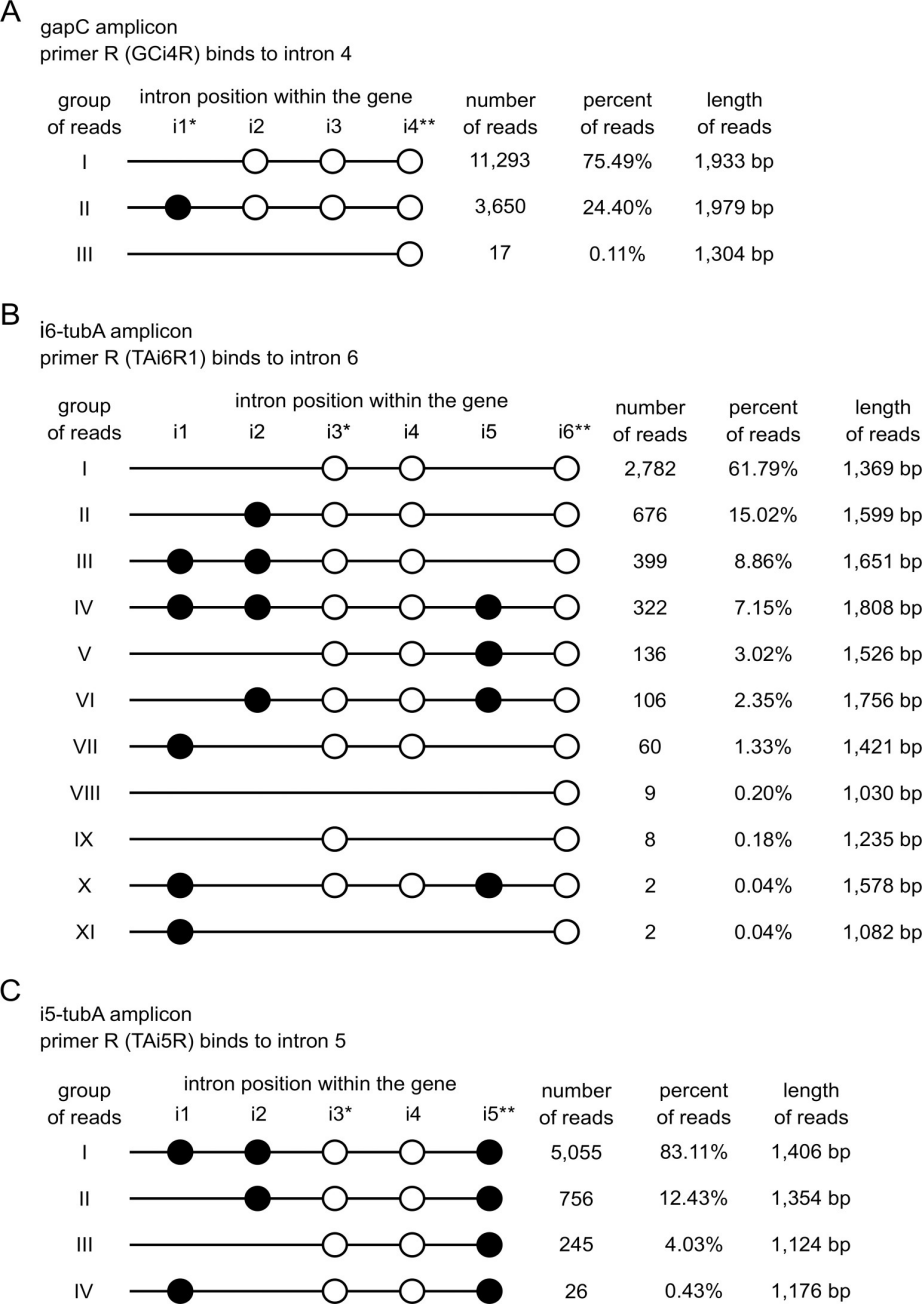
Categories of sequencing reads inferred from the PacBio NGS sequencing. (A) Categories of sequencing reads deriving from the gapC amplicon, (B) i6-tubA amplicon and (C) i5-tubA amplicon, respectively. Number and percentage of particular types of reads are given. The distribution of introns in all groups of reads is depicted. Lines represent the exons while circles represent the introns: black circles: conventional introns; white: nonconventional ones (not to scale). Introns are labelled i1 –i6. In addition, introns that exhibit intermediate features are marked with an asterisk. Double asterisk indicates the introns to which the reverse primer was targeted, thus not considered in the analysis of amplicons (introns to which primers annealed were inevitably present in the amplification products).

In order to analyze splicing intermediates of the *tubA* and *gapC* transcripts, as well as to define the general order of splicing, single molecule real time sequencing of the three different PCR amplicons was performed: (1) amplicon obtained in reaction with primers complementary to the first exon and the last, fourth intron (nonconventional) in the *gapC* gene, using the cDNA from the reverse transcription reaction, with the primer complementary to the last, fourth intron as template, (Fig 3A; amplicon gapC), (2) amplicon obtained in the reaction with primers complementary to the first exon and the sixth, last intron in the *tubA* gene (nonconventional intron), using the cDNA from reverse transcription reaction with the primer complementary to the sixth, last intron as template (Fig 3B; amplicon i6-tubA), (3) amplicon obtained in reaction with primers complementary to the first exon and the fifth, penultimate intron in the *tubA* gene (conventional intron), using the cDNA from the reverse transcription reaction with the primer complementary to the fifth intron as template (Fig 3C; amplicon i5-tubA). The PCR products obtained, corresponding only to the splicing intermediates (where the respective intron–last or penultimate–has been maintained), were sequenced using the PacBio platform.

### Summarized statistics of sequencing reads

As a result of sequencing, raw PacBio reads of various lengths corresponding to three abovementioned amplicons were received. In total, 29,593 reads were obtained for the gapC, i6- and i5-tubA amplicons. After mapping according to the reference sequences and manual examination of contigs, about 4,049 unreliable reads for those three amplicons were discarded (in sum). Thus, 25,544 credible sequencing reads, fulfilling the criteria of complete amplification length were selected for further analysis. All of the accepted reads for *gapC* (14,960), as well as for i6- (4,502) and i5- (6,082) *tubA* were successfully assigned to particular categories based on the alignments with the appropriate reference sequences.

### Categories of sequencing reads

The gapC amplicon produced three possible categories of sequencing reads, with: (I) the first conventional intron absent, (II) all introns present, and (III) all analyzed introns spliced out. Reads representing the first type were the most abundant and accounted for 75.49% (11,293 reads) of the total pool of accepted sequences. The second category was represented by 24.40% (3,650) of reads, whilst only 0.11% (17) of reads was assigned to the third–the least abundant class. Among 14,960 analyzed sequences, reads with the conventional intron present, but with the nonconventional introns absent, were not found (Fig 3A).

In the case of reads deriving from the primer complementary to the sixth, last intron in the *tubA* gene (i6-tubA amplicon), 11 categories of splicing intermediates were distinguished (Fig 3B). Over half of these products (61.79%, 2,782) was represented by reads (I) without the conventional introns, albeit with the nonconventional ones maintained. Another fraction of reads consisted of sequences with: (II) two conventional introns (i1 and i5) spliced out (15.02%, 676), (III) only the fifth conventional intron (i5) removed (8.86%, 399), and (IV) all introns present (7.15%, 322). By contrast, the three following categories of reads occurred in fairly low abundances, in which: (V) the first and the second conventional introns (i1 and i2) were spliced out (3.02%, 136), (VI) only the first conventional intron (i1) was missing (2.35%, 106) and (VII) the second and the fifth conventional introns (i2 and i5) were absent (1.33%, 60). The least frequent were the sequencing reads in which (VIII) all analyzed introns were absent (0.20%, 9), (IX) only the third and the sixth nonconventional introns (i3 and i6) were present (0.18%, 8), (X) only the second conventional intron (i2) was absent (0.04%, 2) and (XI) the first conventional intron (i1) were retained (0.04%, 2).

Sequencing reads deriving from the primer complementary to the fifth intron in the *tubA* gene (i5-tubA amplicon) were divided into four categories (Fig 3C). The most abundant group (83.11%, 5,055) consisted of (I) full-length reads (with all introns present). Another group was formed by (II) the sequences lacking the first conventional intron (12.43%, 756). A relatively small group consisted of reads in which (III) the first and the second conventional introns (i1 and i2) were missing (4.03%, 245). Finally, a very limited fraction was represented by reads with (IV) only the second conventional intron missing (0.43%, 26).

## Discussion

### Conventional introns are removed before nonconventional ones

The results of the pairwise comparisons of the relative order of intron removal are consistent for all examined conventional/nonconventional intron pairs (Fig 1E and 1F; pairs B, D, E and F). On this basis it can be assumed, that the conventional introns in the *gapC* and *tubA* transcripts are removed before the nonconventional ones. The analysis of splicing intermediates confirms such assumption. The results obtained for the gapC and i5-tubA amplicons are unambiguous. Considering the thousands of reads analyzed, if at least one conventional intron is present, nonconventional introns are also present (total no. of reads: 9,487, gapC: 3,650, i5-tubA: 5,837; Fig 3A and 3C). On the other hand, in all transcripts with any nonconventional intron removed, the conventional ones are also absent (total: 17, gapC: 17, i5-tubA: 0; Fig 3A and 3C). Similar results were obtained for i6-tubA amplicon: 1701 reads contain at least one conventional intron and all nonconventional ones. In turn, 17 reads correspond to transcripts with at least one nonconventional intron removed and without conventional ones (Fig 3B). However, in the case of the i6-tubA amplicon, two reads, which are incompatible with the presented results, were found. While the reads from group XI (Fig 3B) harbor the first (i1, XI) conventional intron, they also lack two nonconventional introns (i3 and i4). There are two possible explanations for this phenomenon: (1) reads correspond to conventionally, yet improperly spliced transcripts (although no mutations at crucial positions were found) and the first intron was retained leading to a nonfunctional mRNA molecule; (2) the observed order of splicing ‘conventional introns first’ is not a rule and there may be some degree of deviation. Considering the huge number of reads confirming the ‘conventional introns first’ principle, the first justification seems to be more likely. The unspliced mRNA molecules might be present even outside the nucleus, as it was shown in earlier studies of hamster and mice cells, where transcripts containing the first intron represented a significant part of the total *aprt* mRNA pool in the cytoplasm [11]. Nonetheless, the second explanation cannot be ruled out.

As mentioned previously, the results presented herein, suggesting that conventional introns are removed before nonconventional ones, contradict the data in the literature [26]. Despite the previous research inclining towards the opposite scenario, the experiment repeated in this study confirmed the ‘conventional introns first’ principle.

### Order of conventional introns removal in *tubA* transcripts

The only example which can allow for tracking the possible order of excision of conventional introns, is provided via the *tubA* gene, where the two conventional introns are located next to each other. Indeed, a comparison of the relative order of their removal does not reveal a clear preference (Fig 1E; pair A). A more straightforward NGS analysis of the splicing intermediates confirms that either of these two introns can be removed as the first. Nonetheless, many more reads correspond to transcripts, from which only the first intron (i1) was removed (Fig 3C; amplicon i5-tubA, groups II and IV– 756 vs. 26 reads). Among the NGS reads representing the transcripts for the i6-tubA amplicon, all possible combinations of occurrence of conventional introns can be noticed: reads with single introns such as i1, i2 or i5 removed (groups VI, X and III; Fig 3B), with pairs of introns removed (i1 and i2, group V; i1 and i5, group II; i2 and i5, group VII; Fig 3B) and reads with all three introns removed (i1 and i2 and i5, group I; Fig 3B). This shows the lack of a strict order for conventional intron removal from the *tubA* transcript and implies a moderate preference for their excision. It seems that intron i5 (157 nt long) is preferentially removed as first (399 reads with i5 primarily removed; Fig 3B, group III), followed by short intron i1, which is 52 nt long (106 reads; Fig 3B, group VI) and finally, the longest of them, intron i2, having 230 nt (only two reads; Fig 3B, group X).

### The pace of nonconventional splicing

The band pattern seen on the agarose gel comparing the relative order of nonconventional intron removal is quite unexpected (Fig 1E and 1F; pairs C, G and H). In all three cases, only the product corresponding to the transcript maintaining both introns was observed, and the product with solely a single intron removed was not detected. Such a picture indicates that the abundance of nonconventional splicing intermediates is very low. Results of sequencing performed for the amplicons confirm this observation. In the case of gapC and i6-tubA, a high level of reads with unprocessed nonconventional introns is present (gapC: 11,293 reads, i6-tubA: 2,782; Fig 3A and 3B). Reads with all analyzed nonconventional introns removed are also detectable (gapC: 17 reads, i6-tubA: 9 reads; Fig 3A and 3B), which suggests that using the primer corresponding to the last intron in the reverse transcription reaction (to exclude mature mRNA from the analysis) was not the main factor limiting the pool of splicing intermediates. However, in this regard results obtained for amplicons should be treated with consideration. Meanwhile, barely eight reads with the single nonconventional intron removed were identified across the studied amplicons (i4 in i6-tubA, group IX; no reads were found without i2 or i3 in gapC and i3 in i6-tubA; Fig 3A and 3B). This number is extremely low when compared to the level of existing intermediate forms of conventional splicing, which were, in fact, very abundant (more than thousands of them among all amplicons) and diversified. The low level of nonconventional splicing intermediates observed in both performed experiments may suggest that nonconventional splicing not only begins later than the conventional one, but also takes place more rapidly. However, the low level of nonconventional splicing intermediates may also be the effect of a coordinated start of the removal process of all nonconventional introns (of quite similar length) from the single transcript.

### Time (localization?) gap between conventional and nonconventional splicing

Multiple types of transcripts at various stages of pre-mRNA maturation were detected as a result of the analysis of the intermediate splicing products. Numerous reads corresponding to immature transcripts containing all introns are observed among the gapC and i6-tubA amplicons. The same applies to reads corresponding to various splicing intermediates as well as reads representing transcripts without analyzed introns. However, a strikingly high percentage of reads equivalent to transcripts with all conventional introns removed and all nonconventional present aroused attention. In the case of the gapC amplicon there are as many as 11,293 of such reads (75.49%), while for the i6-tubA one, 2,782 (61,79%). Though it is worth mentioning that the methodology adopted in the experiment was based on the amplification procedure, implying that the number of obtained reads does not necessarily directly reflect the number of intermediates present in the cell. Nevertheless such a picture may indicate that these types of transcripts accumulate before the dynamic nonconventional splicing begins. This, in turn, may suggest existence of some factor delaying the initiation of nonconventional intron removal. It cannot be ruled out that the different localization of both types of splicing may play a role here. Conventional splicing is tightly coupled with transcription–it starts before a pre-mRNA molecule synthesis completion. Perhaps nonconventional splicing cannot launch at such a stage (maybe due to the lack of splicing factors?) and constitutes a post-transcriptional process, which begins only when the released pre-mRNA molecules, nonetheless lacking conventional introns, reach the appropriate localization in the nucleus. Moreover, it should not be ruled out that nonconventional splicing may take place much later, even in the cytoplasm. However, in order to confirm this hypothesis, more insightful research would have to be done in the future. Unfortunately, due to the lack of a standardized method of investigating transcripts’ localization in *E*. *gracilis* cells, it will be some time before this question is answered.

### Outline

In this article, we summarize a series of experiments determining the order of processing of conventional and nonconventional introns, carried out for *E*. *gracilis*. This constitutes another portion of knowledge about atypical nonconventional introns, a research area still extremely challenging due to the lack of well-established methods. Further studies will be needed to answer some of the remaining questions. What is the mechanism of atypical nonconventional intron removal? Is the theory of their transposon origin correct? Likewise, how may their propagation in nuclear genomes be related to the activity of DNA repair systems (as it was hypothesized previously) [22]. Answers to these matters may allow not only for a better understanding of the sense of maintaining these genetic elements in eugleninds’ and diplonemids’ genomes, but further boost the ability to comprehend/interpret the evolution of other types of intervening sequences.

## Materials and methods

### Cell strain and growth condition

The *Euglena gracilis* strain Z (SAG 1224-5/25) used in this study was originally provided by Culture Collection of Algae at Göttingen University (international acronym: SAG), Germany and maintained at a repository of the Department of Molecular Phylogenetics and Evolution at the University of Warsaw, Poland. Cells were cultured in the Cramer-Myers medium [31], supplemented with 0.8% ethanol and 1% sterilized aqueous soil extract. Cultures were grown photoautotrophically at 18°C under white light exposure (16:8 h light/dark cycle, ca. 27 μmol photons m^-2^ s^-1^ provided by cool white fluorescent tubes). Circa 1 × 10^7^ cells in the logarithmic growth phase were harvested by centrifugation (3000 x g, 5 min, room temperature), and washed twice with 5 ml of nuclease-free water to completely remove the medium residues.

### RNA isolation

Total cellular RNA was extracted immediately from freshly collected cells with the mirVana miRNA Isolation Kit (Invitrogen) according to the manufacturer’s protocol designed for total RNA isolation. The RNA sample was treated with DNase in solution with the DNAfree DNA Removal Kit (Invitrogen) between the organic extraction and transfer onto a glass fiber filter. This procedure was performed according to the rigorous digestion conditions described in the manufacturer's instructions.

### RNA quality and quantity assessment

The purity and concentration of the isolated RNA was examined spectrophotometrically using NanoPhotometer NP80 (Implen) and fluorometrically using Qubit 3.0 (Life Technologies). The integrity of the RNA sample was also checked via electrophoresis under denaturing conditions (0.4 M formaldehyde) and run with a 1x TT buffer (VWR Chemicals), as described in detail by [32].

### Reverse transcription

First strand cDNA was synthesized in a 20 μl reaction mixture containing: 2.5 μg of RNA, reverse transcription primer, 5 mM DTT, 40 U of RiboLock (Thermo Scientific), 0.2 mM dNTPs, 1x reaction buffer (Invitrogen) and 200 U of SuperScript III Reverse Transcriptase (Invitrogen). 10 pmoles gene specific reverse transcription primers were used in cDNA synthesis in order to (1) reinvestigate the maturation of the 5’ end of the *rbcS* transcript (complementary to the intron 1 and exon 2, respectively) [26] and to (2) prepare the tubA and gapC amplicons for NGS (complementary to the last or penultimate intron, respectively). The amount of 0.2 μg of Random Hexamer Primer (Thermo Scientific) was used in cDNA synthesis to determine the relative order of intron removal in the *tubA* and *gapC* genes (sequences of all the primers used in this study are listed in S1 Table). The samples were incubated for 60 min at 55°C. In each case, negative controls which consisted of reaction mixtures without the addition of a reverse transcriptase, were also performed.

### PCR amplification

Products of cDNA synthesis were used as a template in PCR reactions carried out as follows. A standard 25 μl reaction mixture contained 0.5 U of Phusion High-Fidelity DNA Polymerase (Thermo Scientific), 0.2 mM dNTPs, 10 pmol of each primer, 1x reaction buffer HF (Thermo Scientific), 1.2 μg of Taq Single-Stranded DNA Binding Protein (EURx) and 1 μl of the first strand cDNA or 1 μl of the negative control reaction from the previous step. 36 cycles of PCR were performed. After an initial 30 s at 98°C, each cycle consisted of 10 s denaturation at 98°C, 15 s annealing at a temperature optimal for each primer set (S1 Table) and primer extension at 72°C for 30 s– 1 min 30 s (depending on expected product sizes). The final extension step was performed for 5 min at 72°C. PCR products were sized on 1.5% - 3% agarose gels and visualized by Midori Green (Nippon Genetics) staining.

### Purification of PCR products and Sanger sequencing

Amplified DNA fragments intended for NGS (gapC, i6- and i5-tubA amplicons) were purified directly from the solution with the GeneMATRIX PCR/DNA Clean-Up Purification Kit (EURx), according to the manufacturer’s instructions. PCR products intended for Sanger sequencing (all products obtained in pairwise comparison of splicing of adjacent introns in *gapC*, *tubA* and *rbcS* genes–as shown in Fig 1, Fig 2) were extracted from gel slices using the QIAEX II Gel Extraction Kit (Qiagen) according to the manufacturer’s instructions. Samples were further sequenced directly from both strands using the BigDye Terminator Cycle Sequencing Ready Reaction Kit 1.1 (Thermo Scientific) and primers specific for each product. Obtained DNA sequences were assembled in SeqMan Pro 14 software (Lasergene, DNA Star) and aligned to the appropriate sequences of *E*. *gracilis*, retrieved from the GenBank database (*gapC* gene accession number: L39772.1; *tubA* gene accession number: AF182557.1).

### Single molecule real time sequencing and data analysis

The amplicons obtained as a result of RT-PCR reactions for the *gapC* and *tubA* genes were sequenced commercially in the Museum and Institute of Zoology of the Polish Academy of Science, Warsaw, Poland, on the PacBio RS II (Pacific Biosciences) instrument. Sequencing reads were initially quality controlled and trimmed by the sequencing institution. In addition, a FastQC 0.11.7 [33] analysis was performed in order to ensure whether the preliminary data quality assessment was carried out correctly. Afterwards, sequencing reads were filtered manually, based on the sequences of primers used for amplification. Subsequently, reads corresponding to the *gapC* and *tubA* genes respectively were saved in separate FASTQ files and converted to FASTA format using the Geneious Basic 10.2.2 software [34]. All reads were then assembled in SeqMan Pro 14 software (Lasergene, DNA Star). Each of the *gapC* and *tubA* contigs was mapped using Geneious mapper algorithm against the reference sequences of *gapC* (GenBank accession number: L39772.1) and *tubA* (GenBank accession number: AF182557.1) genes of *E*. *gracilis*, respectively. The following mapping parameters were applied: medium-low sensitivity/fast mapping with multiple best matches set to all, finding short insertions and large deletions up to 1000 bp as well as including insertions in structural variants, minimum support for structural variant discovery = 2; without trimming. Only successfully mapped, complete reads–flanked by PCR primers at both ends (which indicates complete PCR product amplification) were taken into consideration in the further analysis. Incomplete reads (without the sequence(s) of primers), as well as reads extending beyond the primer binding site(s) were discarded. Reads containing ambiguous nucleotides (mismatches, short insertions or deletions) close to and/or located within the intron/exon junctions were also excluded, as being equivalent to potentially defective products of intron excision. Remaining fair reads were subsequently compared and categorized based on the presence or absence of individual intron(s) in the obtained alignment. Mapped sequences were then sorted based on the alignment length. Afterwards, all possible read types for both studied genes were annotated and counted. The most probable order of intron removal has been inferred from the summarized statistics of the reads.

## Supporting information

S1 Table List of the primers used in this study. Primers used to generate amplicons are marked with an asterisk.
(PDF)

S1 Fig PCR primers used for the pairwise comparison of removal of adjacent introns in *tubA* (A) and *gapC* (C) genes, respectively. Schematic depiction of products obtained with each primer set for both studied genes (B, D). Arrows represent primers, lines represent PCR products. Gaps within spliced products (horizontal dotted lines) refer to the absent intronic sequences. Product lengths are given alongside depictions.
(PDF)

## References

[1] Baurén G, Wieslander L. Splicing of Balbiani ring 1 gene pre-mRNA occurs simultaneously with transcription. Cell. 1994;76: 183–92. 10.1016/0092-8674(94)90182-1 8287477

[2] Moore MJ, Proudfoot NJ. Pre-mRNA processing reaches back to transcription and ahead to translation. Cell. 2009;136: 688–700. 10.1016/j.cell.2009.02.001 19239889

[3] Pandya-Jones A, Black DL. Co-transcriptional splicing of constitutive and alternative exons. RNA. 2009;15: 1896–1908. 10.1261/rna.1714509 19656867 PMC2743041

[4] Singh J, Padgett RA. Rates of in situ transcription and splicing in large human genes. Nat Struct Mol Biol. 2009;16: 1128–33. 10.1038/nsmb.1666 19820712 PMC2783620

[5] Vargas DY, Shah K, Batish M, Levandoski M, Sinha S, Marras SAE, et al Single-molecule imaging of transcriptionally coupled and uncoupled splicing. Cell. 2011;147: 1054–1065. 10.1016/j.cell.2011.10.024 22118462 PMC3245879

[6] Das R, Yu J, Zhang Z, Gygi MP, Krainer AR, Gygi SP, et al SR Proteins Function in Coupling RNAP II Transcription to Pre-mRNA Splicing. Mol Cell. 2007;26: 867–881. 10.1016/j.molcel.2007.05.036 17588520

[7] Neugebauer KM. On the importance of being co-transcriptional. J Cell Sci. 2002;115: 3865–3871. 10.1242/jcs.00073 12244124

[8] Aebi M, Weissman C. Precision and orderliness in splicing. Trends Genet. 1987;3: 102–107. 10.1016/0168-9525(87)90193-4

[9] Bohne J, Wodrich H, Kräusslich HG. Splicing of human immunodeficiency virus RNA is position-dependent suggesting sequential removal of introns from the 5′ end. Nucleic Acids Res. 2005;33: 825–837. 10.1093/nar/gki185 15701754 PMC549389

[10] Attanasio C, David A, Neerman-Arbez M. Outcome of donor splice site mutations accounting for congenital afibrinogenemia reflects order of intron removal in the fibrinogen alpha gene (FGA). Blood. 2003;101: 1851–1856. 10.1182/blood-2002-03-0853 12406899

[11] Kessler O, Jiang Y, Chasin LA. Order of intron removal during splicing of endogenous adenine phosphoribosyltransferase and dihydrofolate reductase pre-mRNA. Mol Cell Biol. 1993;13: 6211–6222. 10.1128/MCB.13.10.6211 8413221 PMC364680

[12] Kim SW, Taggart AJ, Heintzelman C, Cygan KJ, Hull CG, Wang J, et al Widespread intra-dependencies in the removal of introns from human transcripts. Nucleic Acids Res. 2017;45: 9503–9513. 10.1093/nar/gkx661 28934498 PMC5766209

[13] de la Mata M, Lafaille C, Kornblihtt AR. First come, first served revisited: Factors affecting the same alternative splicing event have different effects on the relative rates of intron removal. RNA. 2010;16: 904–912. 10.1261/rna.1993510 20357345 PMC2856885

[14] Adl SM, Simpson AGB, Lane CE, Lukeš J, Bass D, Bowser SS, et al The revised classification of eukaryotes. J Eukaryot Microbiol. 2012;59: 429–93. 10.1111/j.1550-7408.2012.00644.x 23020233 PMC3483872

[15] Mair G, Shi H, Li H, Djikeng A, Aviles HO, Bishop JR, et al A new twist in trypanosome RNA metabolism: cis-splicing of pre-mRNA. RNA. 2000;6: 163–9. 10688355 10.1017/s135583820099229x PMC1369902

[16] Zakryś B, Milanowski R, Karnkowska A. Evolutionary Origin of Euglena. In: Schwartzbach S and Shigeoka S, editors. Euglena: Biochemistry, Cell and Molecular Biology. Adv Exp Med Biol. 2017;979: 3–17. 10.1007/978-3-319-54910-1_1 28429314

[17] de Vargas C, Audic S, Henry N, Decelle J, Mahé F, Logares R, et al Ocean plankton. Eukaryotic plankton diversity in the sunlit ocean. Science. 2015;348: 1261605 10.1126/science.1261605 25999516

[18] Flegontova O, Flegontov P, Malviya S, Audic S, Wincker P, de Vargas C, et al Extreme Diversity of Diplonemid Eukaryotes in the Ocean. Curr Biol. 2016;26: 3060–3065. 10.1016/j.cub.2016.09.031 27875689

[19] Gawryluk RMR, del Campo J, Okamoto N, Strassert JFH, Lukeš J, Richards TA, et al Morphological Identification and Single-Cell Genomics of Marine Diplonemids. Curr Biol. 2016;26: 3053–3059. 10.1016/j.cub.2016.09.013 27875688

[20] Canaday J, Tessier LH, Imbault P, Paulus F. Analysis of Euglena gracilis alpha-, beta- and gamma-tubulin genes: introns and pre-mRNA maturation. Mol Genet Genomics. 2001;265: 153–60. 10.1007/s004380000403 11370862

[21] Milanowski R, Karnkowska A, Ishikawa T, Zakryś B. Distribution of conventional and nonconventional introns in tubulin (α and β) genes of euglenids. Mol Biol Evol. 2014;31: 584–93. 10.1093/molbev/mst227 24296662 PMC3935182

[22] Milanowski R, Gumińska N, Karnkowska A, Ishikawa T, Zakryś B. Intermediate introns in nuclear genes of euglenids–are they a distinct type? BMC Evol Biol. 2016;16: 49 10.1186/s12862-016-0620-5 26923034 PMC4770533

[23] Muchhal US, Schwartzbach SD. Characterization of the unique intron—exon junctions of Euglena gene(s) encoding the polyprotein precursor to the light-harvesting chlorophyll a/b binding protein of photosystem II. Nucleic Acids Res. 1994;22: 5737–5744. 10.1093/nar/22.25.5737 7838730 PMC310141

[24] Russell AG, Watanabe Y, Charette JM, Gray MW. Unusual features of fibrillarin cDNA and gene structure in Euglena gracilis: evolutionary conservation of core proteins and structural predictions for methylation-guide box C/D snoRNPs throughout the domain Eucarya. Nucleic Acids Res. 2005;33: 2781–91. 10.1093/nar/gki574 15894796 PMC1126904

[25] Tessier L, Chart RL, Keller M. Euglena gracilis rbcS. FEBS Lett. 1992;304: 3–6. 10.1016/0014-5793(92)80631-p 1618332

[26] Tessier LH, Paulus F, Keller M, Vial C, Imbault P. Structure and expression of Euglena gracilis nuclear rbcS genes encoding the small subunits of the ribulose 1,5-bisphoshate carboxylase/oxygenase: A novel splicing process for unusual intervening sequences? J Mol Biol. 1995;245: 22–33. 10.1006/jmbi.1994.0003(95)80035-2 7823317

[27] McWatters DC, Russell AG. Euglena Transcript Processing. In: Schwartzbach S and Shigeoka S, editors. Euglena: Biochemistry, Cell and Molecular Biology. Adv Exp Med Biol. 2017;979: 141–158. 10.1007/978-3-319-54910-1_8 28429321

[28] Breckenridge DG, Watanabe Y -i., Greenwood SJ, Gray MW, Schnare MN. U1 small nuclear RNA and spliceosomal introns in Euglena gracilis. Proc Natl Acad Sci. 1999;96: 852–856. 10.1073/pnas.96.3.852 9927657 PMC15314

[29] Ebenezer TE, Carrington M, Lebert M, Kelly S, Field MC. Euglena gracilis Genome and Transcriptome: Organelles, Nuclear Genome Assembly Strategies and Initial Features. In: Schwartzbach S and Shigeoka S, editors. Euglena: Biochemistry, Cell and Molecular Biology. Adv Exp Med Biol. 2017;979: 125–140. 10.1007/978-3-319-54910-1_7 28429320

[30] Ebel C, Frantz C, Paulus F, Imbault P. Trans-splicing and cis-splicing in the colourless Euglenoid, Entosiphon sulcatum. Curr Genet. 1999;35: 542–50. 10.1007/s002940050451 10369962

[31] Cramer M, Myers J. Growth and photosynthetic characteristics of euglena gracilis. Arch Mikrobiol. Springer Nature; 1952;17: 384–402. 10.1007/BF00410835

[32] Mansour FH, Pestov DG. Separation of long RNA by agarose-formaldehyde gel electrophoresis. Anal Biochem. 2013;441: 18–20. 10.1016/j.ab.2013.06.008 23800830 PMC3755752

[33] Barbraham Bioinformatics. FastQC: A quality control tool for high throughput sequence data. [Internet]. 2011 [cited 26 Jan 2018]. Available from: https://www.bioinformatics.babraham.ac.uk/projects/fastqc/

[34] Kearse M, Moir R, Wilson A, Stones-Havas S, Cheung M, Sturrock S, et al Geneious Basic: an integrated and extendable desktop software platform for the organization and analysis of sequence data. Bioinformatics. Oxford University Press; 2012;28: 1647–9. 10.1093/bioinformatics/bts199 22543367 PMC3371832

