# Supplementary material for: Order of removal of conventional and nonconventional introns from nuclear transcripts of Euglena gracilis
Source: PLoS Genet. 2018 Oct 26;14(10):e1007761. doi: 10.1371/journal.pgen.1007761 (PMC6221363; doi:10.1371/journal.pgen.1007761)
Supplement: S1 Table — Primers used to generate amplicons are marked with an asterisk. (PDF) [file pgen.1007761.s001.pdf]

**Supplementary Table S1.** List of the primers used in this study. Primers used to generate amplicons are marked with an asterisk.

| primer ID | Sequence 5'->3' | Tm [ºC] | product ID/pair | intronless product size [bp] | full length product size [bp] |
|---|---|---|---|---|---|
| TAe1F | TGTCCGTCGATTATGGCAAGAAGTCC* | 57 | A1 | 305 | 357 |
| TAi2R | GTGGCAGAGGTCACCAGTCATT | | | | |
| TAi1F | ATCACCTCGTCTTAATGTTTTTTCCATA | 58 | A2 | 242 | 472 |
| TAe3R | ATCGAAGCGCAGAGAAGCCGTCAA | | | | |
| TAe2F | AGCGCCCCACTTACACCAACCTGAAC | 59 | B1 | 234 | 464 |
| TAi3R | CGGCAAACATTTAACAGTCCGAAAGA | | | | |
| TAi2F | TTGGGTATGTCAACTTCGGTCTGG | 58 | B2 | 192 | 397 |
| TAe4R | TGGGCGCATAGGAGGAGAGCAC | | | | |
| TAe3F | CATCTCCTCGTTGACGGCTTCT | 58 | C1 | 402 | 607 |
| TAi4R | AATTTCCTTAACATTTCTGGACAG | | | | |
| TAi3F | TTTGCCGACCTGAAGGGTTTACAGAA | 60 | C2 | 439 | 573 |
| TAe5R | ACGCTGCACCTTGGCCAAATCAC | | | | |
| TAe4F | ACTTCGTGCTCTCCTCCTATGC | 57 | D1 | 458 | 592 |
| TAi5R | AAGCTTGCAAAACTTGGGAAAGA* | | | | |
| TAi4F | AATTTTTGCGAGCTGATTTTCACA | 57 | D2 | 340 | 497 |
| TAe6R | CACCTCCTCGTAGTCCTTCTCCAG | | | | |
| TAe5F | ACTGTTGTCCCTGGTGGTGATTTG | 59 | E1 | 601 | 758 |
| TAi6R | GGGGGTCGGATCGGGTGCTATG | | | | |
| TAi5F | AACACCCACACTACTGAATGACAAAT | 58 | E2 | 321 | 735 |
| TAe7R | AATGAATGCCTTAGCTCTGAACCT | | | | |
| TAi5R(RT) | CACTCCAACAAGCTTGCAAAACTTG* | 55 | i5-tubA | reverse transcription primer | |
| TAi6R1 | TTCGGGCCTGGGTGCATCAAAAA* | 57 | i6-tubA | 1030 - 1756 | 1808 |
| TAi6R(RT) | TGGGGTTAATTCCGTTCAGTTTTCA* | 55 | i6-tubA | reverse transcription primer | |
| GCe1F | CCGTGAAGATTGGCATCAACG* | 60 | F1 | 609 | 655 |
| GCi2R | GCCAAGGAAGCAGGGTCTTGC | | | | |
| GCi1F | CAGGTTCGTGAGTGTCACGT | 60 | F2 | 515 | 766 |
| GCe3R | GTCCATCGACAGTCTTCTGG | | | | |
| GCe2F | GTACGTTGTCGAGTCAACTGG | 60 | G1 | 479 | 730 |
| GCi3R | CAACTGGACAGAATCTCCCAC | | | | |
| GCi2F | CCAAGGTTGTGGGCTTAACG | 60 | G2 | 587 | 965 |
| GCe4R | ACACGGTTGGAGTATCCCCA | | | | |
| GCe3F | CCCTCAAAGAAGGACTGGCGT | 60 | H1 | 696 | 1074 |
| GCi4R | CTTGGTCCCAATGCCAATCACTC* | | | | |
| GCi3F | GCCTGGTTAGGTCCACTGAA | 60 | H2 | 597 | 1038 |
| GCe5R | AGAATCTGGACATGCCGGTT | | | | |
| GCi4R(RT) | ACCTTAGCCCACTGGTCC* | 55 | gapC amplicon | reverse transcription primer | |
| rbcS_1 | GGTGTTTCGTACTGTTGCAG | 58 | 1 - 3 | none | 456 |
| rbcS_2 | CATCTTTGTGGCTCGTTGGA | 59 | 1 - 4 | 494 | 547 |
| rbcS_3(RT) | GAAACCTCTTGCATTTTGAAAA | 61 | SL - 3 | 387 | none |
| rbcS_4(RT) | ACGGCAGCCACCTTAGGC | 60 | SL - 4 | 425 | 478 |
| rbcS_SL | ACTTTCTGAGTGTCTATTTTTTTTCG | 60 | 2 - 3 | none | 287 |
