# Supplementary material for: Order of removal of conventional and nonconventional introns from nuclear transcripts of Euglena gracilis
Source: PLoS Genet. 2018 Oct 26;14(10):e1007761. doi: 10.1371/journal.pgen.1007761 (PMC6221363; doi:10.1371/journal.pgen.1007761)

Supplementary Figure S1. PCR primers used for the pairwise comparison of removal of adjacent introns in tubA(A) and gapC(C) genes, respectively. Schematic depiction of products obtained with each primer set for both studied genes (B, D). Arrows represent primers, lines represent PCR products. Gaps within spliced products (horizontal dotted lines) refer to the absent intronic sequences. Product lengths are given alongside depictions.

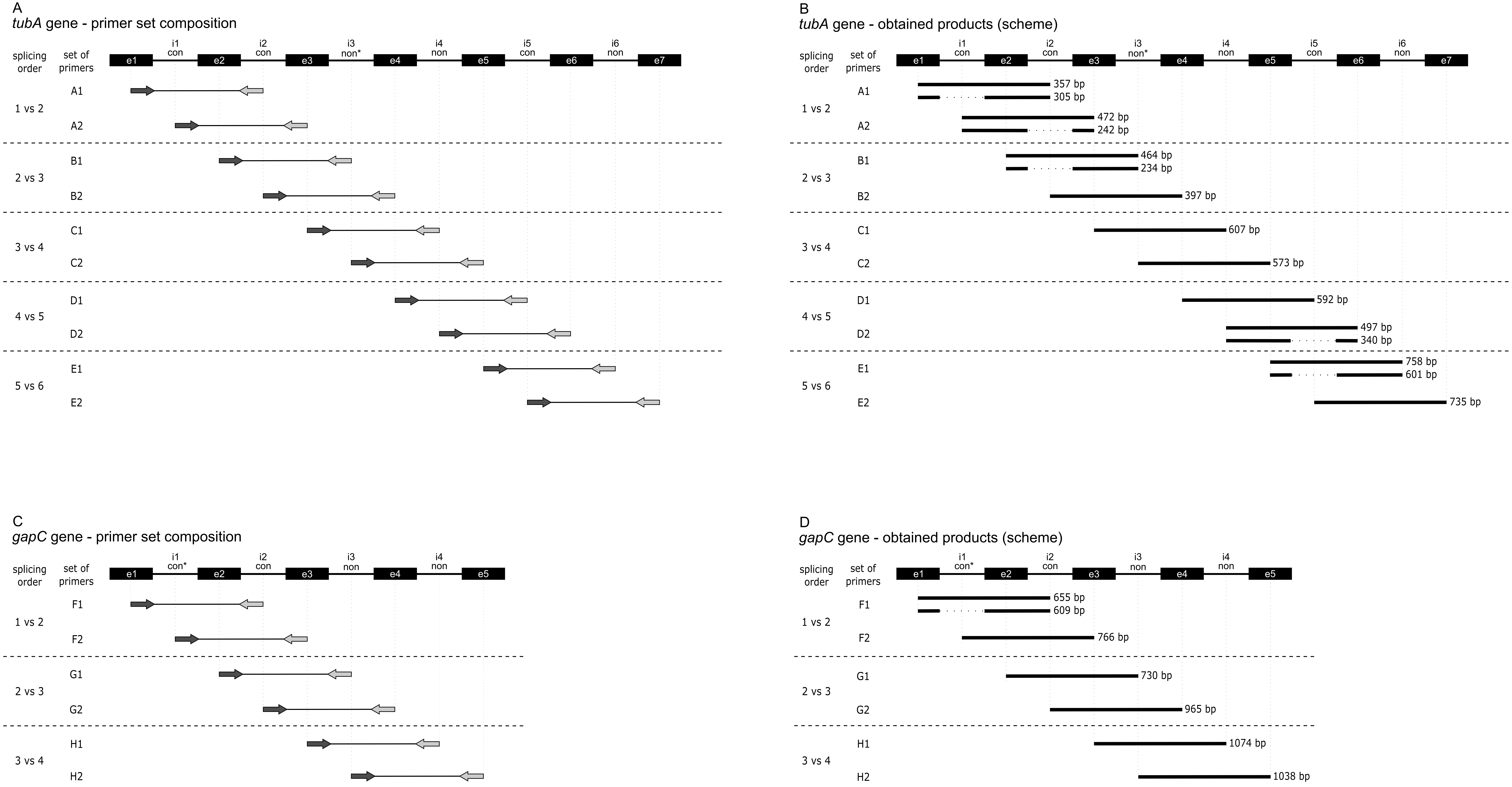

Supplement: S1 Fig — PCR primers used for the pairwise comparison of removal of adjacent introns in tubA (A) and gapC (C) genes, respectively. Schematic depiction of products obtained with each primer set for both studied genes (B, D). Arrows represent primers, lines represent PCR products. Gaps within spliced products (horizontal dotted lines) refer to the absent intronic sequences. Product lengths are given alongside depictions. (PDF) [file pgen.1007761.s002.pdf]
